# Adrenal insufficiency after long-term high-dose ethinylestradiol use in a transgender woman

**DOI:** 10.1210/jcemcr/luag162

**Published:** 2026-06-25

**Authors:** Nagisa Aoki, Tomomi Taguchi, Satoshi Oda, Suguru Tadokoro, Raishi Ichikawa, Takeshi Miyatsuka

**Affiliations:** Department of Diabetes, Endocrinology and Metabolism, Kitasato University School of Medicine, Sagamihara, Kanagawa 252-0374, Japan; Department of Diabetes, Endocrinology and Metabolism, Kitasato University School of Medicine, Sagamihara, Kanagawa 252-0374, Japan; Department of Diabetes, Endocrinology and Metabolism, Kitasato University School of Medicine, Sagamihara, Kanagawa 252-0374, Japan; Department of Diabetes, Endocrinology and Metabolism, Kitasato University School of Medicine, Sagamihara, Kanagawa 252-0374, Japan; Department of Diabetes, Endocrinology and Metabolism, Kitasato University School of Medicine, Sagamihara, Kanagawa 252-0374, Japan; Department of Diabetes, Endocrinology and Metabolism, Kitasato University School of Medicine, Sagamihara, Kanagawa 252-0374, Japan

**Keywords:** adrenal insufficiency, ethinylestradiol, transgender

## Abstract

A 42-year-old transgender woman, assigned male at birth, was referred for the evaluation of hypertension, and was found to have hypercortisolemia with suppressed adrenocorticotropic hormone (ACTH). The patient also presented with obesity and gynecomastia, along with reduced levels of gonadotropins, testosterone, and insulin-like growth factor 1 (IGF-1), with no adrenal lesions on imaging. These findings were attributed to more than 20 years of unsupervised high-dose oral ethinylestradiol use for breast feminization, with doses approximately 4 times higher than those typically prescribed for women. After discontinuation of ethinylestradiol, her hypertension improved, but a rapid ACTH stimulation test demonstrated an inadequate cortisol response, consistent with secondary adrenal insufficiency. Hydrocortisone replacement therapy with gradual tapering led to normalization of her ACTH, cortisol, gonadotropin, and IGF-1 levels. Subsequent ACTH stimulation and corticotropin-releasing hormone tests confirmed the recovery of adrenal function, enabling the cessation of hydrocortisone therapy. Thus, long-term high-dose oral ethinylestradiol may be associated not only with apparent hypercortisolemia attributable to increased corticosteroid-binding globulin, but also, possibly through increased free cortisol, with suppression of the hypothalamic–pituitary–adrenal axis. When a transgender woman taking supraphysiological doses of oral estrogen discontinues estrogen therapy, adrenal insufficiency potentially requiring transient steroid replacement should be considered.

## Introduction

Estrogen agents are known to increase corticosteroid-binding globulin (CBG), resulting in an apparent increase in total cortisol levels without a corresponding increase in biologically active free cortisol [1]. This effect may lead to the overestimation of cortisol action and underdiagnosis of adrenal insufficiency [2]. A previous study reported that a reduction in adrenal hormone levels occurred after the treatment with a gonadotropin-releasing hormone agonist and 17β-estradiol valerate [3]. It remains unknown how the long-term administration of estrogen alone affects adrenal function in individuals assigned male at birth. We here report a case of drug-induced adrenal insufficiency associated with more than 20 years of oral estrogen use in a transgender woman assigned male at birth.

## Case presentation

A 42-year-old Japanese transgender woman, assigned male at birth, presented to a nearby clinic primarily for the evaluation of hypertension, and hypertriglyceridemia was also noted. Her blood pressure was 156/99 mmHg. An evaluation for secondary hypertension revealed a low plasma adrenocorticotropic hormone (ACTH) level of 3.0 pg/mL (SI: 0.66 pmol/L) (reference range: 7.2-63.3 pg/mL [SI: 1.6-13.9 pmol/L]), a high serum cortisol level of 46.6 μg/dL (SI: 1286 nmol/L) (reference range: 7.07-19.6 μg/dL [SI: 195-541 nmol/L]), a plasma renin activity of 3.6 ng/mL/h (reference range: 0.2-2.3 ng/mL/h in the supine position), and a plasma aldosterone concentration of 81.0 pg/mL (SI: 225 pmol/L) (reference range: 4.0-82.1 pg/mL [SI: 11.1-227.8 pmol/L]). No hydrocortisone injection had been administered at the time of cortisol measurement. She was hence referred to our department for the further evaluation of possible ACTH-independent Cushing syndrome.

## Diagnostic assessment

**Figure 1:**
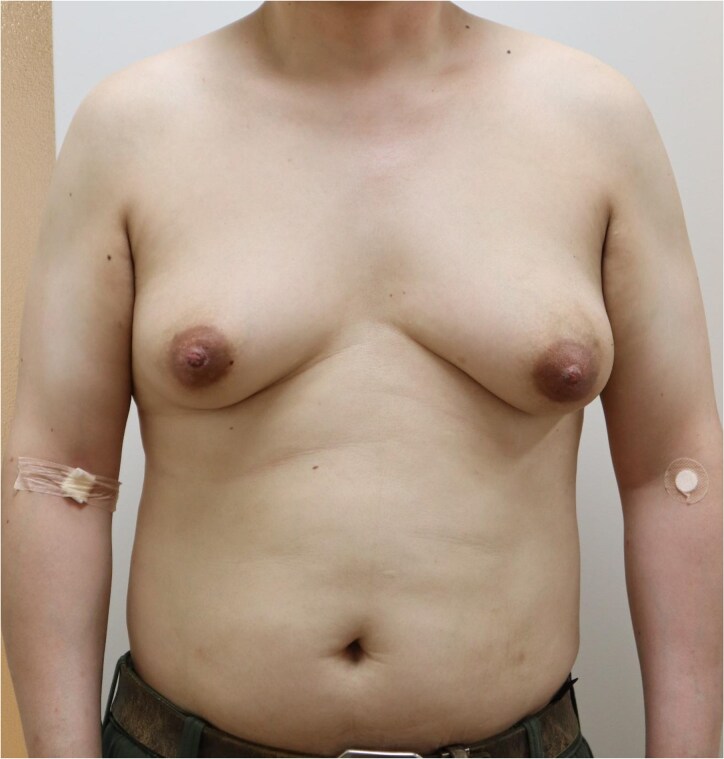
Clinical appearance of bilateral gynecomastia in the patient. Frontal view of the chest demonstrating marked bilateral gynecomastia in the patient before discontinuation of high-dose ethinylestradiol.

The patient occasionally used diflucortolone valerate ointment on a limited area of the dorsum of her hand to treat allergic contact dermatitis caused by rubber gloves. The ointment was used only intermittently, and she was not using it at the time of the evaluation. Physical examination revealed obesity and gynecomastia (Fig. 1). Cushing signs were not observed. Fasting hormonal testing (Table 1) showed low plasma ACTH (7.0 pg/mL [SI: 1.54 pmol/L]), suppressed gonadotropins (luteinizing hormone (LH) < 0.10 IU/L, reference range 1.7-8.6 mIU/mL [SI: 1.7-8.6 IU/L]; follicle-stimulating hormone (FSH) < 0.05 mIU/mL, reference range 1.5-12.4 mIU/L[SI: 1.5-12.4 IU/L]), low total testosterone (< 0.03 ng/mL [SI: < 0.10 nmol/L], reference range 1.31-8.71 ng/mL [SI: 4.54-30.20 nmol/L]), and low insulin-like growth factor 1 (IGF-1) (38 ng/mL [SI: 5.0 nmol/L], reference range 93-259 ng/mL [SI: 12.2-33.9 nmol/L]). Whereas the patient's serum cortisol level was increased (45.6 μg/dL [SI: 1258 nmol/L]) in the absence of hydrocortisone injection, dehydroepiandrosterone sulfate was within the reference range (85 μg/dL [SI: 2.31 μmol/L], reference range 70-495 μg/dL [SI: 1.90-13.43 μmol/L]). The patient's 24-hour urinary free cortisol was not measured as she was unable to complete urine collection. Her estradiol level was suppressed (<5.1 pg/mL [SI: <18.7 pmol/L]; reference range, 14.6-48.8 pg/mL [SI: 53.6-179.1 pmol/L]), likely reflecting negative feedback from long-term high-dose ethinylestradiol use, resulting in suppression of endogenous estradiol production. Gynecomastia in this case was likely attributable to the estrogenic effects of exogenous ethinylestradiol. Computed tomography showed liver steatosis, and neither an adrenal tumor nor adrenal atrophy was observed.

**Table 1:** Laboratory data

	Before discontinuation of ethinylestradiol	4 weeks after discontinuation of ethinylestradiol	6 months after discontinuation of ethinylestradiol	Reference range
WBC	9700 /μL [9.7×10^9^/L]	9400 /μL [9.4×10^9^/L]	7300 /μL [7.3×10^9^/L]	3,300-8,600 /μL [3.3-8.6×10^9^/L]
Neut	73.4% [73.4%]	58.4% [58.4%]	64.6% [64.6%]	50.0-70.0% [50.0-70.0%]
Eosin	1.9% [1.9%]	2.8% [2.8%]	2.7% [2.7%]	2.0-5.0% [2.0-5.0%]
Lym	19.40% [19.40%]	33.1% [33.1%]	26.7% [26.7%]	20.0-40.0% [20.0-40.0%]
Mono	4.8% [4.8%]	5.3% [5.3%]	5.5% [5.5%]	3.0-6.0% [3.0-6.0%]
Baso	0.5% [0.5%]	0.4% [0.4%]	0.5% [0.5%]	0.2-1.0% [0.2-1.0%]
Sodium	137 mEq/L [137 mmol/L]	140 mEq/L [140 mmol/L]	138 mEq/L [138 mmol/L]	138-145 mEq/L [138-145 mmol/L]
Potassium	3.8 mEq/L [3.8 mmol/L]	3.7 mEq/L [3.7 mmol/L]	4.1 mEq/L [4.1 mmol/L]	3.6-4.8 mEq/L [3.6-4.8 mmol/L]
Chloride	102 mEq/L [102 mmol/L]	103 mEq/L [103 mmol/L]	101 mEq/L [101 mmol/L]	101-108 mEq/L [101-108 mmol/L]
ACTH	7.0 pg/mL [1.54 pmol/L]	3.4 pg/mL [0.75 pmol/L]	8.6 pg/mL [1.89 pmol/L]	7.2-63.3 pg/mL [1.59-13.94 pmol/L]
Cortisol	45.6 μg/dL [1258 nmol/L]	3.37 μg/dL [93 nmol/L]	10.2 μg/dL [281 nmol/L]	7.07-19.6 μg/dL [195-541 nmol/L]
DHEA-S	85 μg/dL [2.31 μmol/L]	8 μg/dL [0.22 μmol/L]	44 μg/dL [1.19 μmol/L]	93-259 μg/dL [2.52-7.03 μmol/L]
LH	< 0.10 mIU/mL [< 0.10 IU/L]	2.45 mIU/mL [2.45 IU/L]	5.97 mIU/mL [5.97 IU/L]	0.79-5.72 mIU/mL [0.79-5.72 IU/L]
FSH	< 0.05 mIU/mL [< 0.05 IU/L]	3.08 mIU/mL [3.08 IU/L]	8.48 mIU/mL [8.48 IU/L]	2.00-8.30 mIU/mL [2-8.3 IU/L]
Testosterone	< 0.03 ng/mL [< 0.1 nmol/L]	0.23 ng/mL [0.8 nmol/L]	0.22 ng/mL [0.76 nmol/L]	1.31-8.71 ng/mL [4.54-30.2 nmol/L]
Estradiol	< 5.1 pg/mL [< 18.7 pmol/L]	< 5.1 pg/mL [< 18.7 pmol/L]	< 5.1 pg/mL [< 18.7 pmol/L]	< 5.1 pg/mL [< 18.7 pmol/L]
TSH	1.66 μIU/mL [1.66 mIU/L]	0.94 μIU/mL [0.94 mIU/L]	1.04 μIU/mL [1.04 mIU/L]	0.50-5.00 μIU/mL [0.5-5 mIU/L]
Free T4	1.24 ng/dL [16 pmol/L]	1.16 ng/dL [14.9 pmol/L]	1.34 ng/dL [17.2 pmol/L]	0.90-1.70 ng/dL [11.6-21.9 pmol/L]
GH	0.12 ng/mL [0.12 μg/L]	0.12 ng/mL [0.12 μg/L]	0.11 ng/mL [0.11 μg/L]	< 2.47 ng/mL [< 2.47 μg/L]
IGF-1	38 ng/mL [5 nmol/L]	70 ng/mL [9.2 nmol/L]	89 ng/mL [11.7 nmol/L]	70-495 ng/mL [9.2-64.8 nmol/L]
PRL	23 ng/mL [23 μg/L]	9.6 ng/mL [9.6 μg/L]	8.04 ng/mL [8.04 μg/L]	4.29-13.69 ng/mL [4.29-13.69 μg/L]

Abbreviations: ACTH, adrenocorticotropic hormone; Baso, basophil; DHEA-S, dehydroepiandrosterone sulfate; Eosin, eosinophil; FSH, follicle-stimulating hormone; GH, growth hormone; IGF-1, insulin-like growth factor 1; LH, luteinizing hormone; Lym, lymphocyte; Mono, monocyte; Neut, neutrophil; PRL, prolactin; TSH, thyroid-stimulating hormone; WBC, white blood cell.

Reference range of DHEA-S is that of individuals aged 41-50 years.

Reference range of IGF-1 is that of individuals aged 42 years.

At the second visit, the patient revealed that she identified as a transgender woman and had been taking ethinylestradiol at a dose of 0.05 mg 4 times daily for more than 20 years to achieve breast feminization, which is approximately 4 times higher than the dose typically prescribed for women to treat menstrual irregularities. As her long-term ethinylestradiol use was suspected to be contributing to her hypercortisolism, hypogonadotropic hypogonadism, and low IGF-1 levels, she was ordered to discontinue ethinylestradiol. After discontinuation of ethinylestradiol, the patient's high total cortisol level rapidly decreased, and the rapid ACTH stimulation test (tetracosactide acetate 250 mcg, intravenous bolus) showed a baseline cortisol level of 3.37 μg/dL (SI: 93.0 nmol/L) and a peak cortisol level of 8.55 μg/dL (SI: 236.0 nmol/L), indicating adrenal insufficiency.

## Treatment

At the time of ethinylestradiol discontinuation, hydrocortisone was initiated at a dose of 30 mg/day as glucocorticoid replacement for secondary adrenal insufficiency and was gradually tapered over more than 1 year. After discontinuation of ethinylestradiol, the serum cortisol level decreased from 12.7 μg/dL (SI: 350 nmol/L) at 2 weeks to a nadir of 3.37 μg/dL (SI: 93.0 nmol/L) at 4 weeks, followed by a slight increase to 4.33 μg/dL (SI: 119 nmol/L) at 11 weeks. She did not receive gender-affirming surgery or hormonal therapy, and declined testosterone replacement therapy despite relative testosterone deficiency.

## Outcome and follow-up

**Figure 2:**
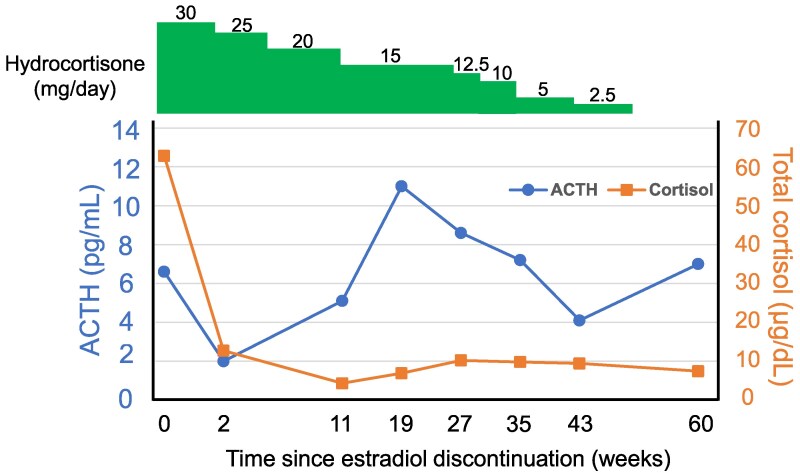
Time course of hydrocortisone replacement therapy, and adrenocorticotropic hormone (ACTH) and total cortisol levels of the patient. Week 0 indicates the time of discontinuation of ethinylestradiol use and initiation of hydrocortisone replacement therapy. Morning ACTH and total cortisol levels are shown over time together with the hydrocortisone dose.

After the discontinuation of ethinylestradiol, the patient frequently experienced fatigue and irritability, which coincided with biochemical hypocortisolemia, and her blood pressure decreased. Hydrocortisone was tapered cautiously over more than 1 year because of concerns about possible steroid withdrawal syndrome and incomplete clinical recovery. During the tapering period, she occasionally complained of reduced concentration. With the tapering of hydrocortisone, the patient's morning ACTH and cortisol levels gradually returned to the normal range (Fig. 2), and the IGF-1 level also recovered to 89 ng/mL (SI: 11.7 nmol/L) (Table 1). A second rapid ACTH stimulation test was performed 6 months after discontinuation of hydrocortisone and demonstrated a baseline cortisol level of 9.79 μg/dL (SI: 270 nmol/L) and a recovered peak cortisol level of 26.2 μg/dL (SI: 723 nmol/L). The corticotropin-releasing hormone test showed baseline ACTH and cortisol levels of 4.1 pg/mL (SI: 0.90 pmol/L) and 9.41 μg/dL (SI: 260 nmol/L), respectively, with peak values of 26.8 pg/mL (SI: 5.90 pmol/L) and 19.20 μg/dL (SI: 530 nmol/L), respectively.

Six months after discontinuation of ethinylestradiol, LH, FSH, and testosterone levels increased to 5.97 mIU/mL (SI: 5.97 IU/L), 8.48 mIU/mL (SI: 8.48 IU/L), and 0.22 ng/mL (SI: 0.76 nmol/L), respectively, suggesting partial recovery of the HPG axis. However, testosterone levels remained low, indicating insufficient functional recovery. No further hormonal treatment was initiated because the patient did not wish to resume gender-affirming hormonal therapy and also declined testosterone replacement therapy. Gynecomastia decreased slightly but persisted throughout the observation period.

## Discussion

Various studies have demonstrated the effects of estrogen on circulating corticosteroid levels. Women treated with oral contraceptive steroids have been shown to have lower ACTH levels and higher cortisol levels than untreated women [4]. These estrogen-associated changes may be related to increased hepatic synthesis of CBG and, possibly, to changes in free cortisol that could contribute to suppression of the hypothalamic–pituitary–adrenal (HPA) axis. Levels of free cortisol can be assessed by measuring salivary and urinary cortisol [5], although these measurements were not performed in the present case. A recent study showed that oral estrogen use may affect not only total cortisol through increased CBG but also free cortisol to a lesser degree [6]. Although the estrogen exposure in that study was equivalent to ethinylestradiol 30 mcg/day, the present patient had been receiving a much higher dose of ethinylestradiol approximately 120 mcg/day, which may have resulted in a greater increase in free cortisol, suppression of the HPA axis, and the development of secondary adrenal insufficiency after discontinuation of ethinylestradiol. Importantly, the discontinuation of ethinylestradiol resulted in a rapid decrease in cortisol level and a blunted cortisol response to ACTH stimulation, indicating secondary adrenal insufficiency. This temporal sequence, with hypercortisolemia during exposure and hypocortisolemia after withdrawal, raises the possibility of estrogen-associated suppression of the HPA axis and subsequent secondary adrenal insufficiency after discontinuation. In previous case reports measuring ACTH and cortisol levels during estrogen therapy or oral contraceptive use, comorbid conditions that could affect these measurements were common, which confounded the accurate assessment of adrenal function. Furthermore, heterogeneity in the duration of estrogen exposure in these previous studies precluded direct comparison with our case. Moreover, to our knowledge, no studies to date have performed dynamic stimulation testing both during estrogen treatment and after its discontinuation for the detailed evaluation of adrenal function and to longitudinally monitor adrenal recovery over an extended period (Table 2) [7  8  9  10  11  12]. Only one case has been reported in which adrenal insufficiency was diagnosed in a patient by dynamic stimulation testing, but as adrenal insufficiency did not improve after the discontinuation of estrogen therapy, it remains unclear whether the condition was induced by sex hormone treatment [7]. In the present report, the patient's cortisol levels were clearly increased during ethinylestradiol use, followed by hypocortisolemia after discontinuation, with the subsequent gradual recovery of ACTH and cortisol levels over time. Although these findings do not establish causality, the temporal association and reversibility suggest that ethinylestradiol may have contributed to suppression of the HPA axis and the subsequent development of secondary adrenal insufficiency.

**Table 2:** Comparison of endocrine function during and after estrogen therapy between previously reported patients and the present patient

Reference	Age, sex*	Comorbidity	During estrogen therapy				After estrogen therapy			
			ACTH	Cortisol	Rapid ACTH stimulation test	Urine cortisol	ACTH	Cortisol	Rapid ACTH stimulation test	Urine cortisol
Iida et al [7]	59-year-old, male	Prostate cancer	Low	Low	Poor response	Low	—	—	—	—
Anno et al [8]	31-year-old, male	None	Normal	High	—	—	Normal	High	—	—
Okuno et al [9]	27-year-old, female	Adrenalectomy for CS	Low	High	—	—	Normal	Normal	—	—
Bando et al [10]	53-year-old, male	Adrenal incidentaloma	Normal	High	—	—	—	Normal	—	Normal
Yu [11]	50-year-old, female	Familial dysalbuminemia	Normal	High	—	Low	Normal	High	—	—
Lewandowski et al [12]	20-year-old, female	Addison disease	Low	High	—	—	High	Low	—	—
Present study	42-year-old, male	None	Low	High	Poor response	—	Normal	Normal	Normal response	**—**

Results are expressed as categorical interpretations based on the institutional reference ranges: “Low,” below the lower limit of normal; “Normal,” within the reference range; and “High,” above the upper limit of normal. “–” denotes not performed.

^*^Sex refers to biological sex as reported in the original studies.

Abbreviations: ACTH, adrenocorticotropic hormone; CBG, corticosteroid-binding globulin; CS, Cushing syndrome.

High-dose oral ethinylestradiol may also have affected the growth hormone (GH)–IGF-1 axis in the present case. Oral estrogen is known to attenuate GH action mainly in the liver, thereby reducing circulating IGF-1 levels [13]. Furthermore, in this case, oral estrogen may have contributed to suppression of the GH–IGF-1 axis through an increase in free cortisol, resulting in secondary GH deficiency. Therefore, the patient's fatigue may have reflected not only secondary adrenal insufficiency but also, at least in part, concomitant functional GH deficiency. Furthermore, the gradual recovery of the GH–IGF-1 axis after discontinuation of ethinylestradiol suggests that improvement in fatigue may also have paralleled this endocrine recovery.

Some transgender women who are assigned male at birth and identify as women often undergo estrogen replacement therapy and/or surgery to achieve a more feminine body shape [14]. In the present case, we explained the option of breast augmentation surgery to the patient, as discontinuation of estrogen therapy may lead to a reduction in breast volume. However, she did not wish to undergo surgical intervention. Hormone therapy for transgender women confers many of the same risks as sex hormone replacement therapy in non-transgender individuals. Although the guidelines for hormone therapy in gender dysphoria recommend periodic monitoring of prolactin levels and venous thromboembolism in transgender women receiving estrogen therapy, they do not address the potential risk of adrenal insufficiency [15]. In this present case, the patient self-administered estrogen at supratherapeutic doses, which likely placed her at a markedly increased risk of various adverse effects.

In conclusion, discontinuation of long-term supraphysiological ethinylestradiol intake may unmask secondary adrenal insufficiency. Therefore, the possibility of adrenal insufficiency should be considered in transgender women with long-term supraphysiological use of feminizing hormones, and adrenal function should be evaluated both before and after the discontinuation of estrogen therapy.

## Learning points

- Long-term oral estrogen use at supraphysiological doses can increase total cortisol and suppress ACTH levels, thereby resulting in laboratory findings that mimic ACTH-independent Cushing syndrome.
- Prolonged supraphysiological estrogen use may lead to secondary adrenal insufficiency, particularly after its discontinuation.
- When caring for transgender women taking supraphysiological doses of oral estrogen long term, the evaluation of adrenal function should be considered, particularly when they discontinue estrogen therapy.

## Data Availability

Original data generated and analyzed during this study are included in this published article.

## Abbreviations

ACTH: adrenocorticotropic hormone
CBG: corticosteroid-binding globulin
FSH: follicle-stimulating hormone
HPA: hypothalamic–pituitary–adrenal
IGF-1: insulin-like growth factor 1
